# Abdomen (A Pandora’s Box): A Delayed Presentation of Blunt Abdominal Injury With a Mesenteric Tear Leading to Gangrenous Bowel

**DOI:** 10.7759/cureus.67002

**Published:** 2024-08-16

**Authors:** Abishek Prasenaa, Thinagaran K, Reegan Jose, Karthick G

**Affiliations:** 1 General Surgery, SRM Medical College Hospital and Research Centre, Kattankulathur Campus, Chengalpattu, IND

**Keywords:** case report, mesenteric tear, gangrenous bowel, blunt abdominal trauma, peritonitis, laparotomy

## Abstract

In the emergency department, blunt trauma to the abdomen is frequently seen following a blunt injury. Road traffic accidents are the leading cause of this type of injury. This is a case presentation of a young adult male who presented to the emergency medicine department one week after he had a road traffic accident. After the initial assessment, an ileal perforation with peritonitis was suspected, and the patient was taken up for surgery. Intraoperative findings led to the patient being diagnosed with a case of gangrenous ileum secondary to a complete mesenteric tear. This case report highlights how a high level of suspicion and prompt intervention in a timely manner can play an important role in reducing morbidity and case fatality.

## Introduction

Blunt abdominal injury followed by blunt trauma is a common presentation in the emergency department (ED). The most common cause is road traffic accidents, followed by falls, sports injuries, and physical assaults. Blunt trauma to the abdomen can occur in individuals of all age groups. Intra-abdominal injury following blunt abdominal trauma is 12-15% [1]. Intestinal and mesenteric injuries are less common than solid organ injuries. In abdominal traumas, hollow organ and mesenteric injuries are found in 3-5 % of patients. When we consider mesenteric injuries, the mesentery of the small bowel is injured more frequently than that of the colon and it has a high case fatality rate [2]. This diagnosis delay is also caused by the decreasing number of exploratory laparotomy, due to the development of imaging methods capable of identifying lesions that are conservatively managed. However, certain lesions such as mesenteric lacerations are missed [1].

The authors present the case of a 38-year-old man with gangrenous bowel secondary to complete mesenteric tear who had a blunt abdominal injury one week prior and who walked into the ED as a late presentation.

## Case presentation

A 38-year-old male, with no comorbidities, presented as a walk-in to the ED with an alleged history of road traffic accident, a skid, and a fall from his two-wheeler, one week ago. The patient received first aid in a nearby primary health center and went to his residence since he thought he only had superficial injuries. The patient developed abdominal pain three days after the injury for which he did not seek any medical advice. Subsequently, abdominal pain increased in severity with vomiting for which he came to the ED as a walk-in. The patient had a complaint of abdominal pain, which was diffuse in nature, dull aching type, continuous in nature, and non-radiating with no aggravating or relieving factors. The patient also had complaints of vomiting for two days, multiple episodes per day, non-projectile, contained food particles, non-blood stained, and non-bilious. The patient also had a positive history of hiccoughs, obstipation, loss of appetite, headaches.

On examination, the patient was conscious, oriented, and afebrile, no pallor, icterus, cyanosis, clubbing, pedal edema, and generalized lymphadenopathy. The patient was tachycardic, tachypneic, and normotensive and was maintaining oxygen saturation above 98% on room air. Per abdomen findings, the inspection showed a distended abdomen with no visible peristaltic movements. On palpation, there was diffuse tenderness in all quadrants, a single site of maximum tenderness could not be elicited, and a positive rebound tenderness and guarding were present in the right upper and lower quadrants. There was no rigidity and no woody abdomen; percussion of the abdomen gave resonant notes and absent bowel sounds on auscultation. Rectal findings showed no boggy swelling in the anterior rectal wall region. Other systemic examinations were unremarkable. Extended Focused Assessment with Sonography for Trauma (E-Fast) showed the presence of free fluid in the abdomen. With the above-mentioned history and examination findings, a working diagnosis of blunt injury abdomen with hollow viscous perforation with no signs of sepsis was made and the patient was planned for emergency laparotomy and proceeded. The patient's blood investigations before taking up for surgery are shown in Table 1.

**Table 1:** Blood investigations of the patient before taking up for surgery g/dl: grams per deciliter, cumm: cubic millimeter

Parameter	Value	Reference range
Hemoglobin	10.2 g/dl	13-17 g/dl
White blood cell count	13,000/cumm	4000-11000/cumm
Platelet count	3,71,000/cumm	1,50,000-4,50,000/cumm

**Figure 1:**
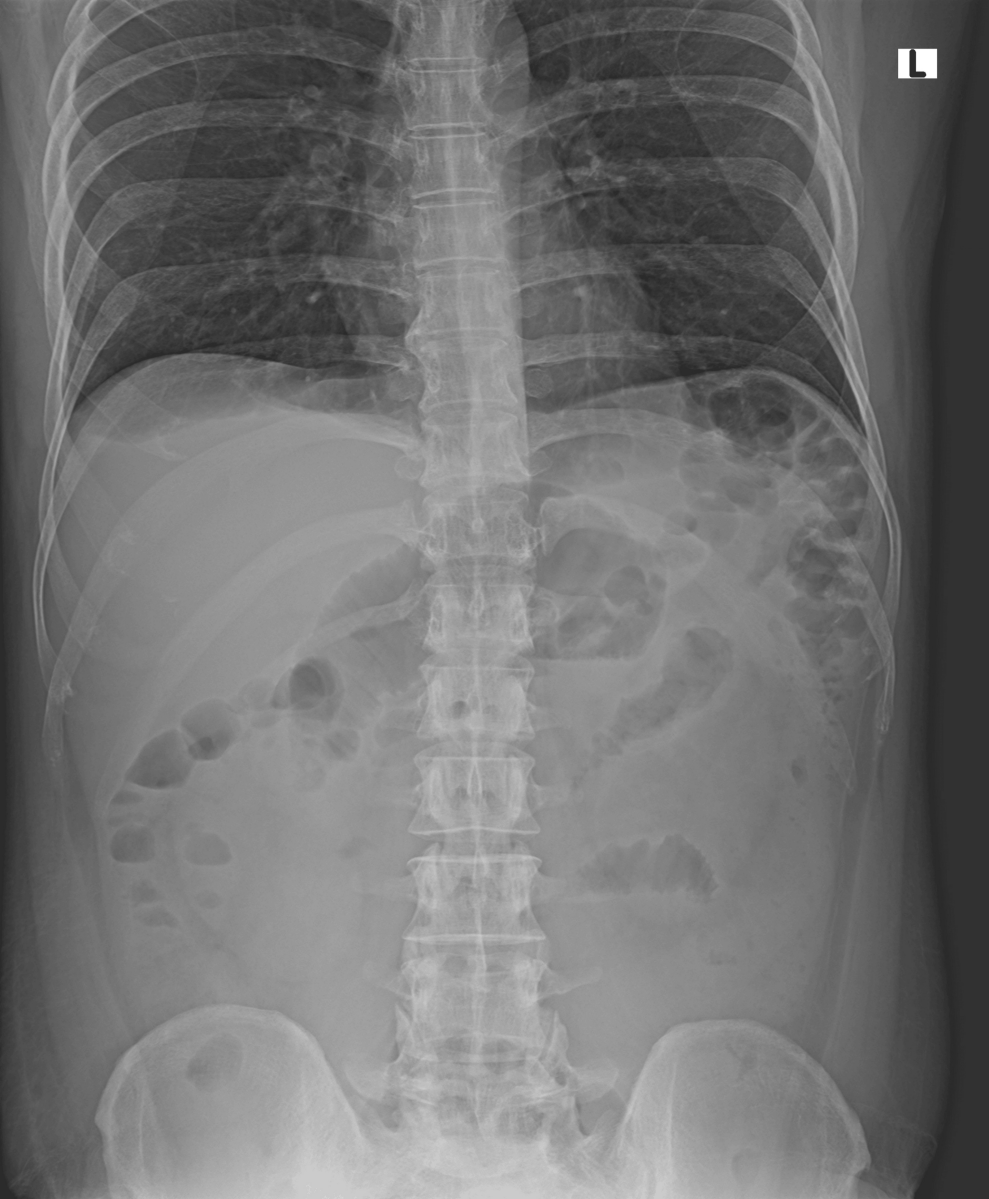
X-ray abdomen-erect showing nonspecific changes

**Figure 2:**
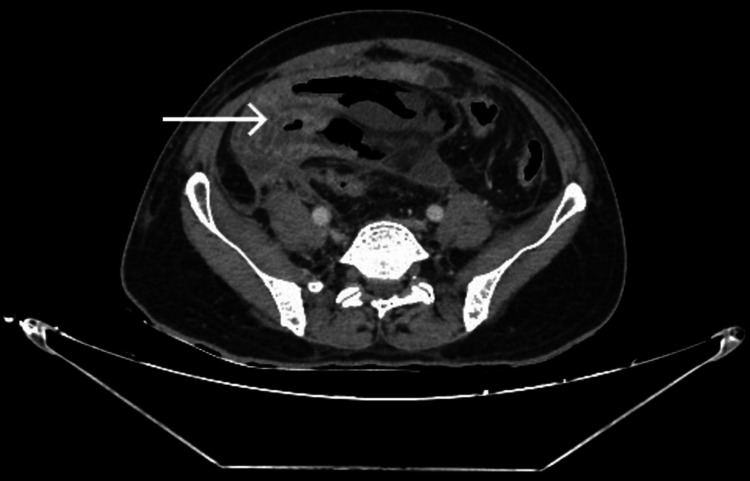
CECT abdomen axial view showing thickened ileal segments CECT: contrast enhanced computed tomography

**Figure 3:**
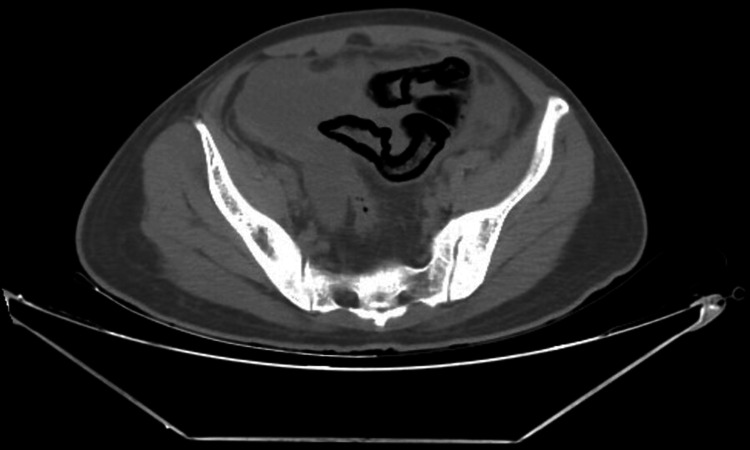
CECT abdomen axial view showing pneumatosis intestinalis CECT: contrast-enhanced computed tomography

X-ray abdomen-erect showed few air-fluid levels in the small bowel, as shown in Figure 1, which was nonspecific and not contributary to the final diagnosis. Contrast enhanced computed tomography (CECT) of the abdomen showed a partly well-defined thin-walled, approximately two mm in thickness, fluid collection measuring 8.9 mm x 9.5 mm x 6.2 mm (anteroposterior x transverse x craniocaudal) involving the right iliac fossa, which is seen to have suspicious communication with the distal ileal loop. There is a long-segment hypo-enhancing wall thickening noted involving the distal and terminal ileal loops for a length of approximately 20 cm showing a maximum thickness of 3.5 mm with diffuse adjacent fat stranding and fluid as shown in Figure 2. Pneumatosis intestinalis, which is air inside the bowel walls, i.e., intramural, was seen, as shown Figure 3. Fluid was noted in the peri/subhepatic bilateral paracolic gutter and peri-splenic region with diffuse mesenteric fat stranding.

The patient was taken up for exploratory laparotomy under general anesthesia since laparoscopy was unavailable in the emergency setting. Intraoperative findings revealed the following findings: (i) About 600 ml of straw-colored fluid containing flakes and blood clots was drained. (ii) About 20 cm of the ileum was found to be gangrenous approximately 15 cm from the ileocecal junction. (iii) Complete mesenteric tear of the affected ileal segment was noted. Intraoperatively, the diagnosis of gangrenous ileum secondary to complete mesenteric tear was made. The mechanism of the injury was suspected to be compression and deceleration forces, which caused the mesenteric tear. 

**Figure 4:**
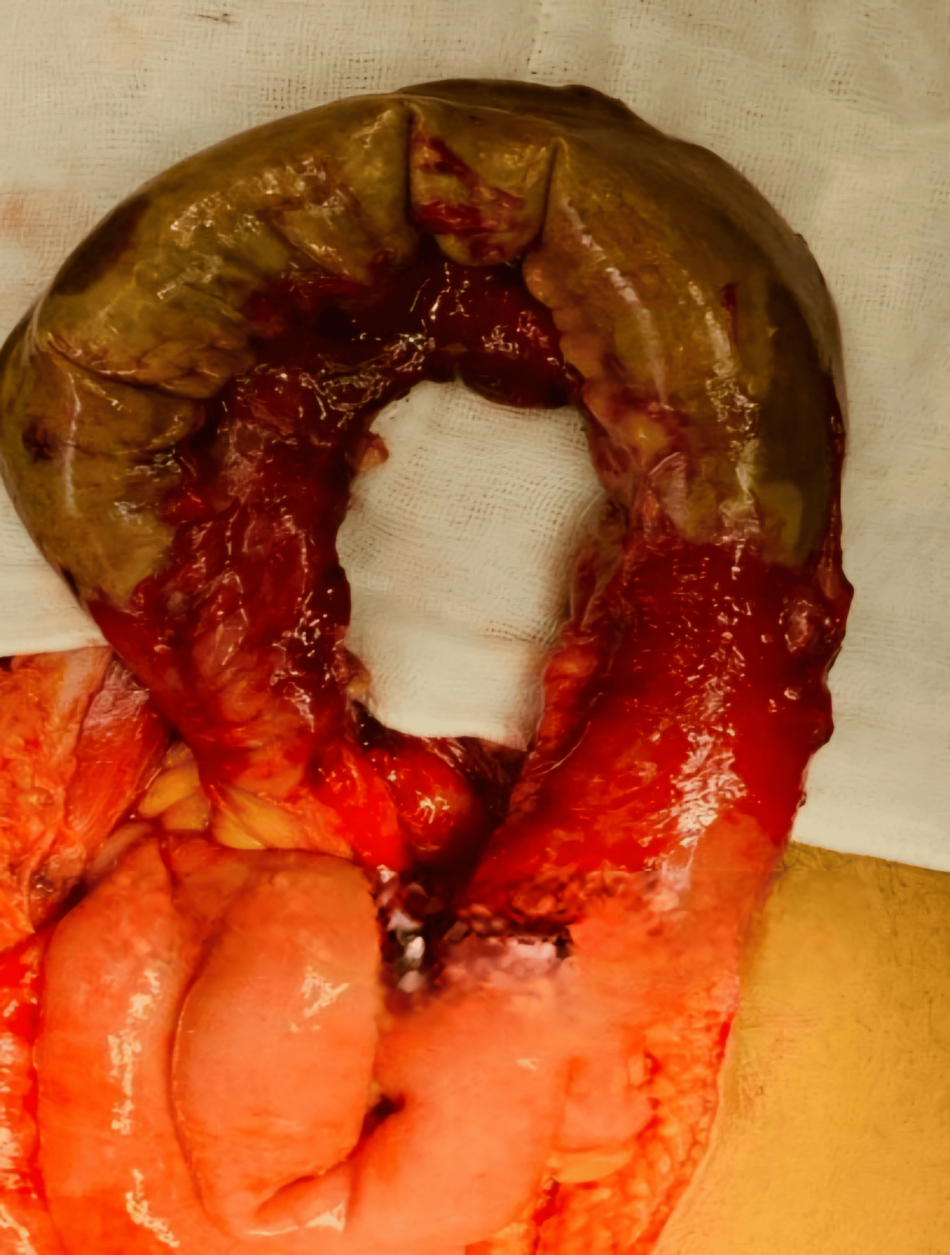
Intraoperative finding of the gangrenous segment of the ileum

**Figure 5:**
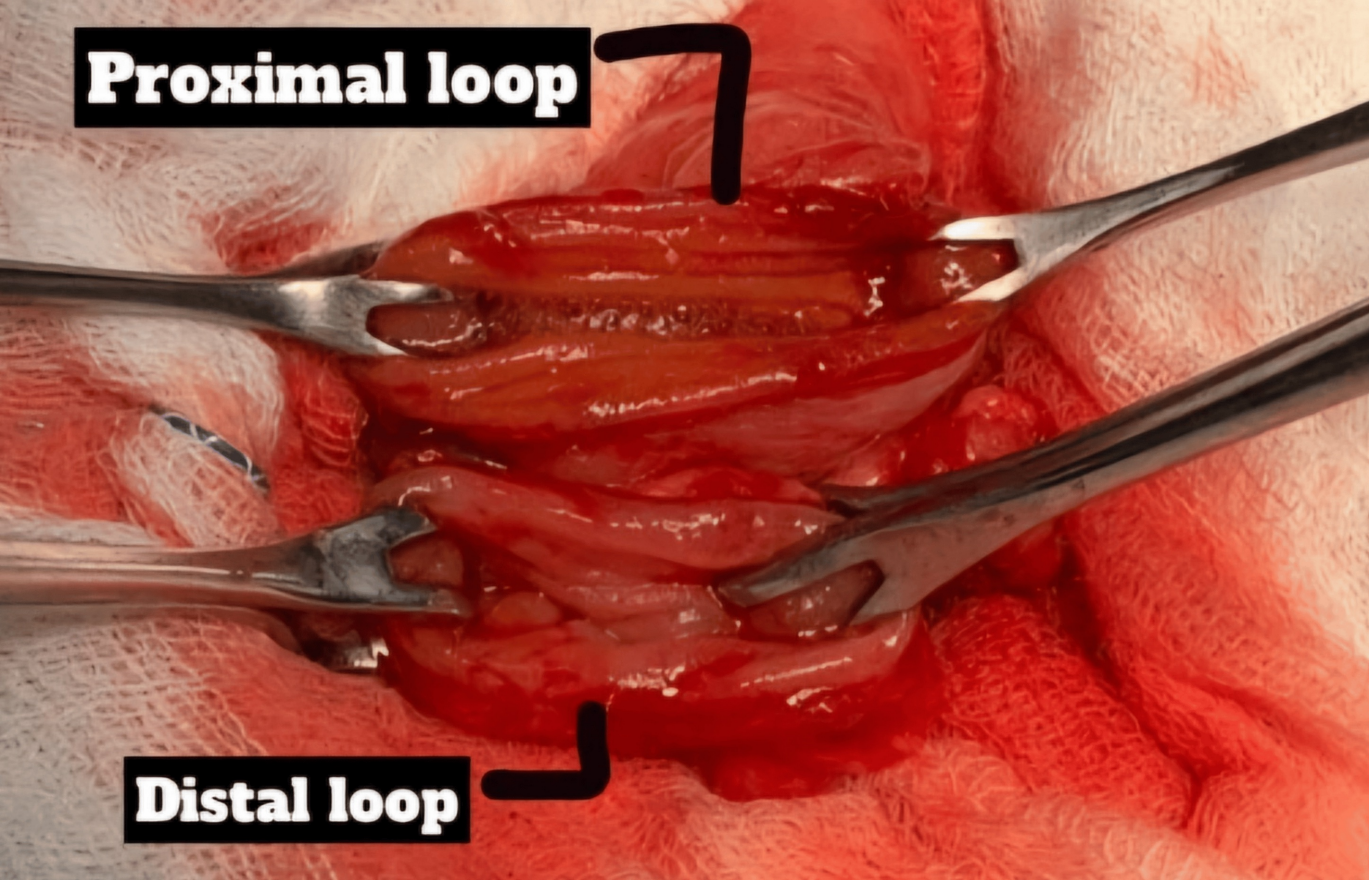
Healthy resected margins of the ileum

**Figure 6:**
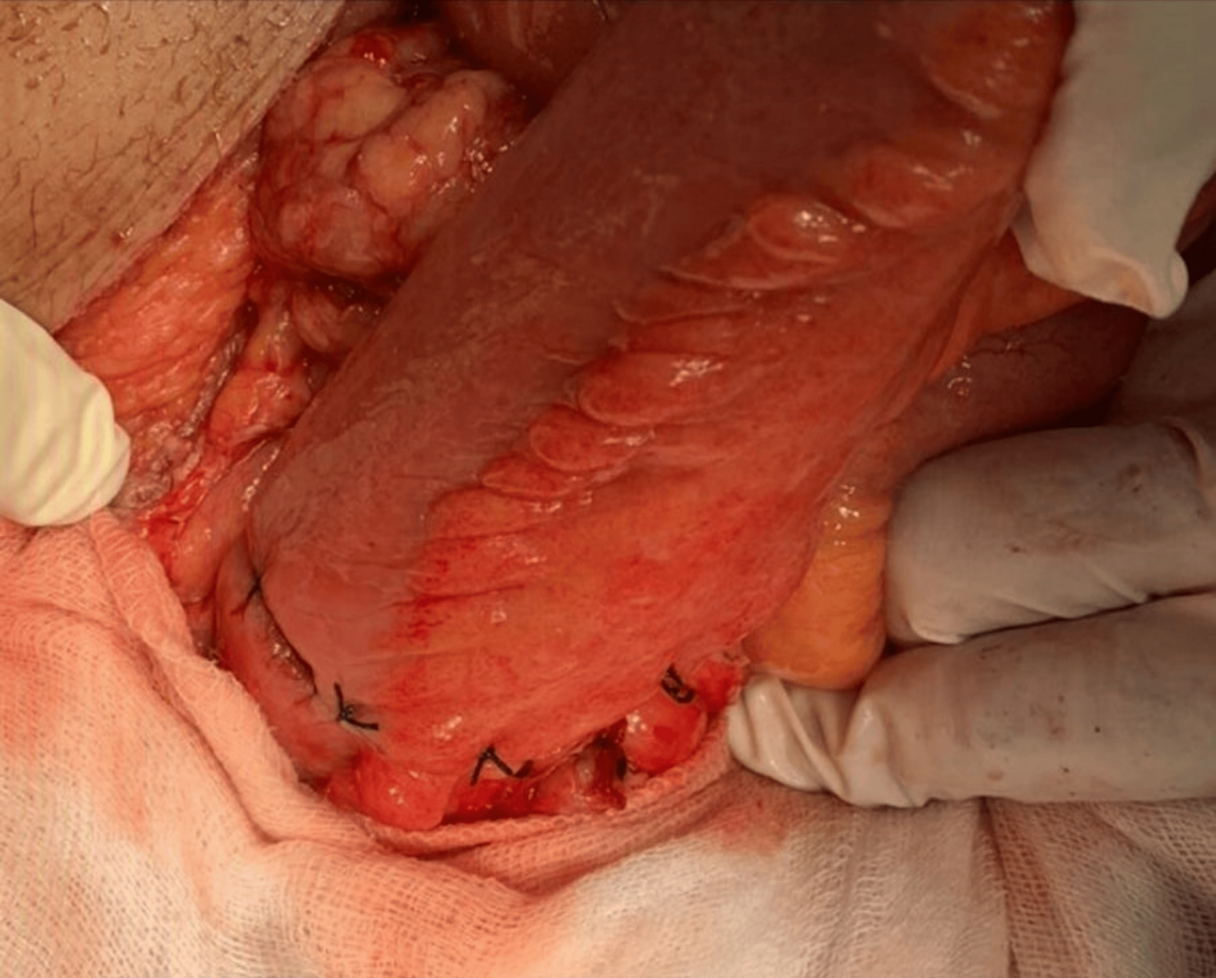
Intraoperative picture showing double-layer ileo-ileal anastomosis

**Figure 7:**
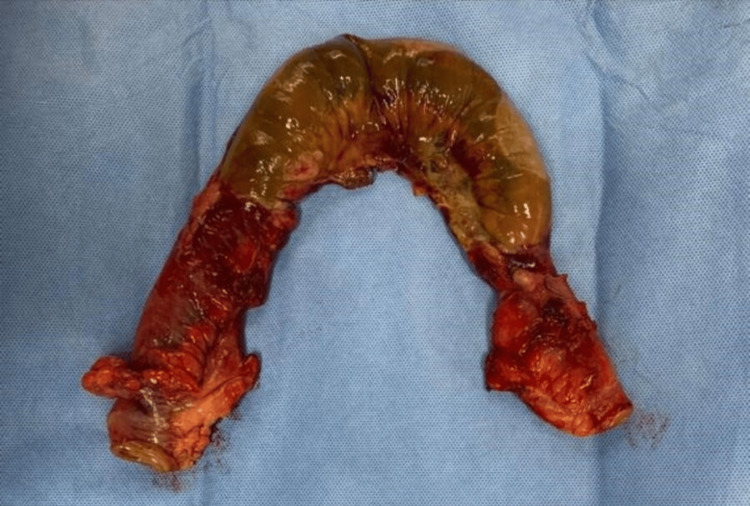
Specimen of the resected gangrenous ileum

The gangrenous ileal segment was resected, and double-layer ileo-ileal anastomosis was done. Intraoperative pictures are shown in Figures 4-7.

Postoperatively, the patient was kept in the post-anesthesia care unit and monitored for four days. The patient was kept nil per oral during this period with adequate intravenous fluids. During the monitoring period, daily blood workups, meticulous input and output chartings, and vitals were recorded. The patient was started on solids by postoperative day six and discharged on postoperative day 10. The patient came for review one week after discharge, and the wound was healthy with no signs of infection or dehiscence. The patient was followed up for two months with no postoperative complications.

## Discussion

Vascular injuries of the abdomen are scarce following blunt injury in comparison to penetrating injuries of the abdomen. Mesenteric injuries resulting from blunt abdominal trauma underscores their rarity but significant potential for morbidity and mortality. These injuries typically stem from high-energy trauma, such as motor vehicle accidents, often affecting the small intestine most frequently, followed by the colon and occasionally the duodenum [2]. Diagnosis poses challenges due to nonspecific symptoms, especially in patients with concurrent injuries like traumatic brain injury or pelvic fractures, and diagnosis is delayed contributing to the high rates as mentioned before and leads to unacceptable diagnostic delays. Surgical intervention based on physical examination has a negative laparotomy rate of 40% [3]. The primary assessment of acute trauma patients involves the standard cABC (cervical spine, airway, breathing, and circulation) protocol [4].

Ultrasound of the abdomen is the investigation of choice in hemodynamically unstable patients. E-FAST can give a clear picture in an acceptable time period and can confirm the presence of intra-abdominal fluid. Hemodynamically stable patients must be subjected to CECT as it is highly sensitive and specific for the diagnosis of mesenteric injuries, revealing signs such as mesenteric hematoma, bowel wall abnormalities, and interloop fluid [4]. However, these injuries may not always be immediately apparent, necessitating a high suspicion, particularly in the presence of abdominal bruising or signs of peritonitis. Further evaluation with a CT scan is needed to delineate the extent of the injury. Management strategies vary based on patient stability and injury severity. Hemodynamically unstable patients require urgent surgical exploration to address bowel ischemia or significant hemorrhage. By contrast, stable patients may initially undergo conservative management or minimally invasive procedures such as laparoscopy, particularly if the injury is localized and not associated with peritonitis [3]. The surgical intervention aims to restore bowel continuity and address mesenteric vascular compromise, with options ranging from resection to bowel repair depending on the extent of injury.

An original article by Gad et al. concluded that road traffic accident is the most common cause of blunt injury to the abdomen with a significant male predominance [5]. In another case report by Ferreira et al., it was shown that the diagnosis was made intraoperatively [6].

The mechanism of injury is compression and deceleration forces, which cause mesenteric tears from the bowel and contribute to the high morbidity and mortality rate [7-8]. Recent advances like transcatheter arterial embolization (TAE) are now being done in selective patients, but due to high rates of failure, surgical management must be kept ready before undergoing the procedure. Moreover, periodical monitoring must be done to identify postoperative complications at the earliest [9].

## Conclusions

The evolution of diagnostic and therapeutic approaches highlights the importance of early recognition and intervention in improving outcomes for patients with mesenteric injuries. Surgical management remains the best and gold-standard intervention for the diagnosis and treatment of mesenteric tear secondary to blunt injury to the abdomen. Continued research is necessary to refine diagnostic criteria and treatment algorithms, particularly in integrating newer technologies and approaches such as TAE for stable patients. Overall, high suspicion, prompt diagnosis, and appropriate management remain pivotal in reducing the morbidity and mortality associated with mesenteric injuries from blunt abdominal trauma.
